# Family physicians’ knowledge, attitudes, and practices regarding complementary and alternative medicine in Saudi Arabia: an exploratory cross-sectional study

**DOI:** 10.3389/frhs.2026.1837145

**Published:** 2026-10-07

**Authors:** Safaa M. Alsanosi, Asim M. Alshanberi, Aljawharah Alqathama, Majd Almech, Husna Irfan Thalib, Bandar M. Abuageelah, Ameena Manzoor, Ruwaydah Hamoud M. Alruways, Yosra Alhindi, Maram H. Alshareef

**Affiliations:** 1 Department of Pharmacology and Toxicology, Faculty of Medicine, Umm Al-Qura University, Makkah, Saudi Arabia; 2 General Medicine Practice Program, Batterjee Medical College, Jeddah, Saudi Arabia; 3 Pharmaceutical Sciences Department, Faculty of Pharmacy, Umm Al-Qura University, Makkah, Saudi Arabia; 4 General Medicine Practice Program, Batterjee Medical College, Aseer, Saudi Arabia; 5 Department of Community Medicine and Pilgrims Health Care, Faculty of Medicine, Umm Al-Qura University, Makkah, Saudi Arabia

**Keywords:** attitudes, complementary medicine, family physicians, herbal medicine, primary care

## Abstract

**Introduction:**

The use of complementary and alternative medicine (CAM) is increasing worldwide, driven by cultural, religious, and socioeconomic factors, as well as its holistic approach to health. While previous Saudi and regional studies have explored healthcare professionals’ views on CAM, recent data on family physicians in Saudi Arabia are limited. This exploratory study examines their knowledge, attitudes, and practices regarding CAM.

**Methods:**

An exploratory cross-sectional study was conducted among family physicians in Saudi Arabia using an interviewer-administered questionnaire from 1 August 2025 to 31 January 2026. Data were analysed using descriptive statistics, with associations between categorical variables assessed using the chi-square or Fisher's exact tests and statistical significance set at *p* < 0.05

**Results:**

Of the 62 family physicians surveyed in Saudi Arabia, 58.1% were aged 36–45 years, and the gender distribution was equal. The majority (66.1%) held consultant positions, 40.3% had 5–10 years of experience, and only 32.3% had received formal training in CAM. Overall, knowledge of CAM was moderate. A total of 75.8% supported the integration of CAM into conventional care, while 46.8% expressed uncertainty about the existence of clear national guidelines. Attitudes toward CAM were predominantly positive: 79% supported evidence-based integration, 82.2% endorsed the inclusion of CAM education in undergraduate curricula, and 64.5% agreed that physicians knowledgeable in CAM benefit patients. However, 51.6% perceived CAM as a potential public health concern. In clinical practice, 79% recommended CAM, with meditation (64.5%), herbal supplements (59.7%), spiritual healing (58.1%), and cupping (53.2%) being the most frequently used modalities. Higher levels of CAM knowledge were significantly associated with recommending CAM to a greater proportion of patients (*p* = 0.012).

**Conclusion:**

Family physicians exhibited moderate knowledge of CAM, generally positive attitudes toward evidence-based integration, and selective clinical practices that favored culturally familiar CAM therapies. Additional research and targeted educational initiatives are required to enhance evidence-based CAM practice.

## Introduction

Complementary and alternative medicine (CAM) encompasses a wide range of healthcare practices that fall outside conventional Western medicine, including herbal medicines, acupuncture, chiropractic, spiritual healing, and other traditional interventions (1). Globally, the use of CAM has increased substantially, with a growing proportion of individuals adopting these approaches as complementary or primary treatment options (2). This rising use is driven by a complex interaction of cultural, religious, and socioeconomic factors that shape healthcare behaviours (3). CAM's appeal is frequently attributed to its holistic orientation, which is perceived to address not only physical conditions but also the psychological, emotional, and spiritual aspects of health (4).

Historically, CAM has played an integral role in healthcare systems across many regions, particularly in societies with deeply rooted cultural and religious traditions (5). In recent years, however, interest in CAM has intensified even in settings where conventional healthcare services are widely available (6). This shift reflects increased cultural openness in a globalised context, alongside a growing demand for personalised, patient-centred care. Additionally, preferences for natural and minimally invasive therapies with fewer perceived adverse effects have further contributed to CAM's expanding popularity (7).

The expansion of public interest in and use of CAM has been well documented globally. In many developed countries, approximately half of the population regularly uses at least one form of CAM, including the United States (42%), Australia (48%), France (49%), and Canada (70%) (8–11). This widespread adoption has been accompanied by a parallel increase in CAM-related research and scientific literature, with a growing emphasis on evidence-based approaches (12). In developing countries, CAM is practised both within and outside formal healthcare systems, prompting the development of policy frameworks and preliminary guidelines to support its integration into national healthcare structures (13).

Geographical accessibility may also influence reliance on complementary and alternative medicine. In archipelagic and geographically dispersed settings such as the Philippines, residents of remote island and mountainous communities may experience limited access to physicians and hospital-based services, potentially increasing their reliance on traditional or complementary healthcare practices (9, 10). A study conducted among parents in Cebu City found that perceived healthcare accessibility was associated with attitudes toward CAM, highlighting the interaction between geographical, healthcare-system, and sociocultural factors in shaping CAM utilization (4). Although the healthcare contexts of Saudi Arabia and the Philippines are not directly equivalent, the Philippine experience provides a relevant international example of how geographical barriers and uneven access to conventional healthcare may contribute to CAM reliance (7).

Saudi Arabia exemplifies the global persistence of CAM use, despite significant advancements in healthcare infrastructure, education, and medical technology (14). CAM practices, especially herbal medicine, cupping, and Quranic recitation, remain deeply rooted in cultural and spiritual traditions and are often utilized in conjunction with conventional treatments (15). The ongoing use of these therapies is shaped by cultural and religious beliefs, patient preferences, and the perception that CAM therapies are natural and relatively safe (16–20). Previous research from Saudi Arabia and other Gulf countries has investigated healthcare professionals' knowledge, attitudes, and practices concerning CAM, typically reporting favorable attitudes toward evidence-based integration, variable levels of knowledge, limited formal training, and significant patient interest. However, most available evidence is based on regional and population-specific cross-sectional studies, which restrict methodological consistency and national generalizability (21–24). The current study extends this literature by presenting recent exploratory data from family physicians in Saudi Arabia, with a focus on formal CAM training, counselling confidence, and self-reported recommendation practices.

Clinical practice is influenced not only by knowledge but also by patient expectations, cultural and religious norms, professional regulations, access to evidence, and institutional factors (6, 7). This distinction is especially relevant in the context of CAM, where physicians may discuss or recommend culturally familiar therapies despite limited scientific confidence (11). Additionally, self-reported attitudes and practices may be affected by social desirability bias (25, 26). Integrating CAM into clinical practice in an evidence-informed manner necessitates thorough evaluation of effectiveness, contraindications, product quality, adverse effects, and potential interactions with conventional medicines, rather than uncritical acceptance. This consideration is particularly important for herbal products and supplements, which may pose risks such as herb–drug interactions, inconsistent dosing, contamination, and delays in conventional treatment (18, 21).

Given that family physicians, due to their central role in primary care, medication reconciliation, chronic disease management, and patient counselling, are well positioned to address CAM-related discussions and potential safety concerns. Accordingly, this exploratory study sought to describe CAM-related knowledge, attitudes, and clinical practices among family physicians in Saudi Arabia.

## Methods

### Ethical approval

The study was approved by the Biomedical Research Ethics Committee, Faculty of Medicine, Umm Al-Qura University, Makkah, Saudi Arabia. Approval number HAPO-02-K-012-2024-032080, under the Declaration of Helsinki.

### Study design and eligibility criteria

This exploratory observational study employed a descriptive cross-sectional design to assess family physicians knowledge, attitudes, practices relating to CAM in Saudi Arabia. The study was conducted in primary healthcare centres and clinics between 1 August 2025 and 31 January 2026. Eligible participants were family medicine physicians actively engaged in family medicine or primary care practice, including general practitioners, residents, specialists, and consultants. Participants were required to hold a valid professional licence and to be clinically active during the recruitment period. Physicians were excluded if they were not practising in family medicine or primary care, were not clinically active during the study period, had participated in pilot testing of the questionnaire, or submitted questionnaires with incomplete responses to prespecified essential items.

### Variables and measurement

The questionnaire evaluated four primary domains: participant characteristics, knowledge of complementary and alternative medicine (CAM), attitudes toward CAM, and practices. Each knowledge item received one point for a correct response and zero points for incorrect or ‘not sure’ responses. The total knowledge score was expressed as a percentage of the maximum possible score. Attitudes toward CAM were measured using statements rated on a five-point Likert scale, ranging from 'strongly disagree’ to 'strongly agree.' Negatively worded items were reverse-scored as appropriate. Higher scores reflected more favorable attitudes toward the integration of evidence-based CAM.

The practice domain assessed physicians’ discussions and recommendations of CAM, the modalities most frequently recommended, the frequency of these recommendations, counseling regarding potential harms, and clinical conditions deemed appropriate for CAM use. Multiple responses were permitted where applicable, and conditional questions were analyzed using only eligible participants as the denominator. CAM recommendations were initially assessed as a binary variable and subsequently evaluated as the proportion of patients receiving CAM recommendations, categorized into predefined percentage ranges.

### Data collection instrument

Data collection employed a structured, interviewer-administered questionnaire adapted from previously published instruments that assess healthcare professionals' knowledge, attitudes, and practices regarding complementary and alternative medicine. Content validity was established by four experts in family medicine and clinical pharmacology, who independently evaluated the relevance, clarity, comprehensiveness, and cultural appropriateness of the questionnaire items. The instrument was revised based on their feedback. Subsequently, the questionnaire was pilot-tested among seven family physicians who met the general eligibility criteria but were not included in the final study sample. The pilot assessment examined item clarity, comprehension, administration time, response consistency, and overall feasibility. The complete questionnaire is provided as Supplementary Material S1 to facilitate evaluation and replication.

### Bias mitigation

To minimize interviewer and information bias, all interviews utilized a standardized questionnaire and consistent administration procedures. Interviewers received instructions to read each question verbatim, maintain neutrality, and refrain from prompting participants or offering explanations that might influence responses. Questionnaire items were neutrally phrased and underwent review prior to full administration to enhance clarity and reduce misinterpretation. Participants were informed that participation was voluntary, responses would remain confidential, and neither participation nor refusal would affect employment, professional assessment, or institutional standing. No personally identifiable information was included in the analytical dataset. Individuals involved in pilot testing were excluded from the final study sample. Data coding and entry were verified to minimize transcription and classification errors, and statistical tests were chosen based on variable type and expected cell frequencies.

### Data analysis

All collected data were coded and entered into the Statistical Package for the Social Sciences (SPSS) version 28 (IBM Corp., Armonk, NY, USA) for analysis. Descriptive statistics, including frequencies and percentages, were used to summarise the sociodemographic characteristics, knowledge, attitudes, and practices of healthcare staff regarding CAM. To assess overall knowledge of CAM, practitioners' responses to knowledge-based questions were scored, and participants were classified into three categories: low knowledge (correct responses to less than 30% of the questions), average knowledge (correct responses to 30%–70% of the questions), and good knowledge (correct responses to more than 70% of the questions). Then, those with low and average knowledge were merged into the unsatisfactory knowledge category.

Associations between sociodemographic and professional factors, exposure to CAM training, information sources, and overall knowledge level were examined using the Chi-square test—or Fisher's exact test where expected cell counts were small. Similarly, relationships between overall knowledge level and clinical practice patterns, including patient recommendations, CAM therapy discussions, and perceptions of CAM suitability, were evaluated using appropriate inferential tests. Statistical significance was defined as a *p*-value < 0.05.The study was reported in accordance with the Strengthening the Reporting of Observational Studies in Epidemiology statement for cross-sectional studies. A completed STROBE checklist is provided as Supplementary Material S2.

## Results

A total of 70 physicians were invited to participate. Of these, eight questionnaires were excluded due to incomplete responses. The final analytical sample comprised 62 participants, corresponding to a response rate of 88.6%, as shown in Table 1 more than half of the respondents were aged 36–45 years (58.1%), and gender distribution was equal (50% males and females). As for professional experience, 40.3% of participants had 5–10 years, 35.5% had more than 10 years, and 24.2% had less than 5 years of experience. Most respondents were consultants (66.1%), followed by residents (12.9%), general practitioners (11.3%), and specialists (9.7%). Concerning CAM training exposure, only 32.3% of participants reported having any, whereas more than half of the respondents (53.2%) reported following CAM-related content on social media.

**Table 1:** Sociodemographic and professional characteristics of participating family physicians (*N* = 62).

Socio-Demographic data	*N*	%
Age in years		
25–35	14	22.6%
36–45	36	58.1%
46–55	12	19.4%
Gender		
Male	31	50.0%
Female	31	50.0%
Years of experience		
< 5 years	15	24.2%
5–10 years	25	40.3%
> 10 years	22	35.5%
Professional classification		
General practitioner	7	11.3%
Resident	8	12.9%
Specialist	6	9.7%
Consultant	41	66.1%
CAM training exposure^a^		
Yes	20	32.3%
No	42	67.7%
CAM social media engagement		
Yes	33	53.2%
No	29	46.8%

a Attended lectures, workshops, and certification programs.

Table 2 illustrates participants' knowledge of CAM. Most respondents reported knowing what CAM stands for (85.5%) and having previously heard about it (95.2%). Over half of the participants rated their knowledge as limited (51.6%), while fewer reported excellent knowledge (45.2%). A substantial majority knew that CAM should be an integral part of conventional medicine (75.8%). When asked about the preferred role of CAM, equal proportions viewed it as complementary to conventional medicine and as an integral part of it (35.5%). However, uncertainty was evident regarding regulatory frameworks, as nearly half of the participants (46.8%) were unsure whether clear guidelines for CAM exist in Saudi Arabia.

**Table 2:** Knowledge of family physicians toward CAM (*N* = 62).

Knowledge items	*N*	%
Do you know what CAM stands for?		
Yes	53	85.5%
No	8	12.9%
Not sure	1	1.6%
Have you ever heard of CAM?		
Yes	59	95.2%
No	3	4.8%
Not sure	0	0.0%
How would you rate your knowledge about CAM therapies?		
Limited	32	51.6%
Excellent	28	45.2%
Not sure	2	3.2%
Should CAM be an integral part of conventional medicine?		
Yes	47	75.8%
No	9	14.5%
Not sure	6	9.7%
What CAM should be?		
Complementary to conventional medicine	22	35.5%
An integral part of conventional medicine	22	35.5%
An alternative to conventional medicine	7	11.3%
Not sure	11	17.7%
Should patients be treated exclusively with conventional medicine?		
Yes	26	41.9%
No	32	51.6%
Not sure	4	6.5%
Are there clear guidelines about CAM in Saudi Arabia?		
Yes	13	21.0%
No	20	32.3%
Not sure	29	46.8%

Table 3 shows knowledge and confidence in counselling about widely used and culturally familiar herbs. Ginger was the most well-understood herb, with more than half of participants (59.7%) reporting that they knew its medicinal uses and felt comfortable counselling patients about its benefits and risks. Similar patterns were observed for garlic and cinnamon, with 34 participants (54.8%) reporting confidence in counselling for each. Black seed and turmeric also showed relatively high levels of counselling confidence, by 50% and 46.8% of participants, respectively.

**Table 3:** Knowledge and counselling confidence regarding culturally prevalent herbs among family physicians (*N* = 62).

Herbs	Lack of knowledge and counselling confidence		Knowledge without counselling confidence		Knowledge with counselling confidence	
	*N*	%	*N*	%	*N*	%
Ginger	2	3.2%	23	37.1%	37	59.7%
Garlic	4	6.5%	24	38.7%	34	54.8%
Cinnamon	7	11.3%	21	33.9%	34	54.8%
Fenugreek	32	51.6%	20	32.3%	10	16.1%
Coriander	31	50.0%	20	32.3%	11	17.7%
Ginseng	21	33.9%	31	50.0%	10	16.1%
Echinacea	31	50.0%	24	38.7%	7	11.3%
Ashwagandha	11	17.7%	42	67.7%	9	14.5%
Black seed	10	16.1%	21	33.9%	31	50.0%
Turmeric	15	24.2%	18	29.0%	29	46.8%
Roselle	27	43.5%	9	14.5%	26	41.9%

Figure 1 shows participants’ overall knowledge level of CAM and their main sources of information. Most respondents had an average level of knowledge (59.7%), while over a third had good knowledge (35.5%). Only a small minority showed low levels of knowledge (4.8%). With respect to knowledge sources (Figure 2), peer-reviewed journals were the most frequently reported source (66.1%), followed by the internet (45.2%). Informal sources, such as family and friends, were reported by 14 participants (22.6%), while fewer respondents relied on structured educational pathways, including health databases (19.4%), books (12.9%), and formal courses (9.7%). Other sources were minimally reported (3.2%). For descriptive presentation, knowledge scores were classified as poor (≤39%), fair (40%–69%), or good (≥70%). For the exploratory association analyses presented in Tables 7, 8, the poor and fair categories were combined into a below-good-knowledge category and compared with the good-knowledge category. This dichotomization was based on a previously published KAP scoring convention and was used to reduce sparse contingency-table cells. The threshold was treated as an operational classification and not as a clinically validated measure of CAM competence.

**Figure 1:**
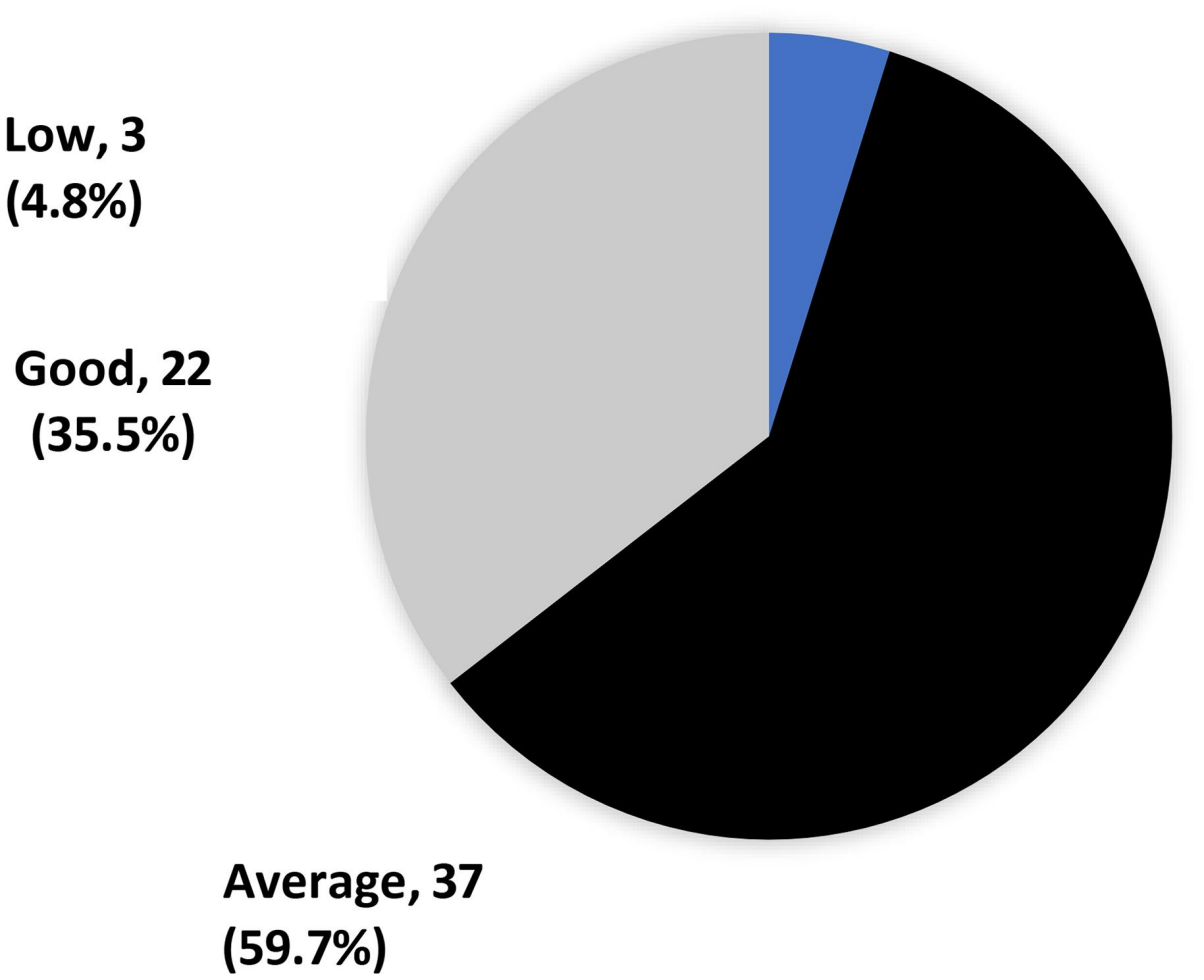
Distribution of CAM knowledge categories among participating family physicians (*N* = 62). Knowledge was categorized as poor (≤39%), fair (40%–69%), and good (≥70%).

**Figure 2:**
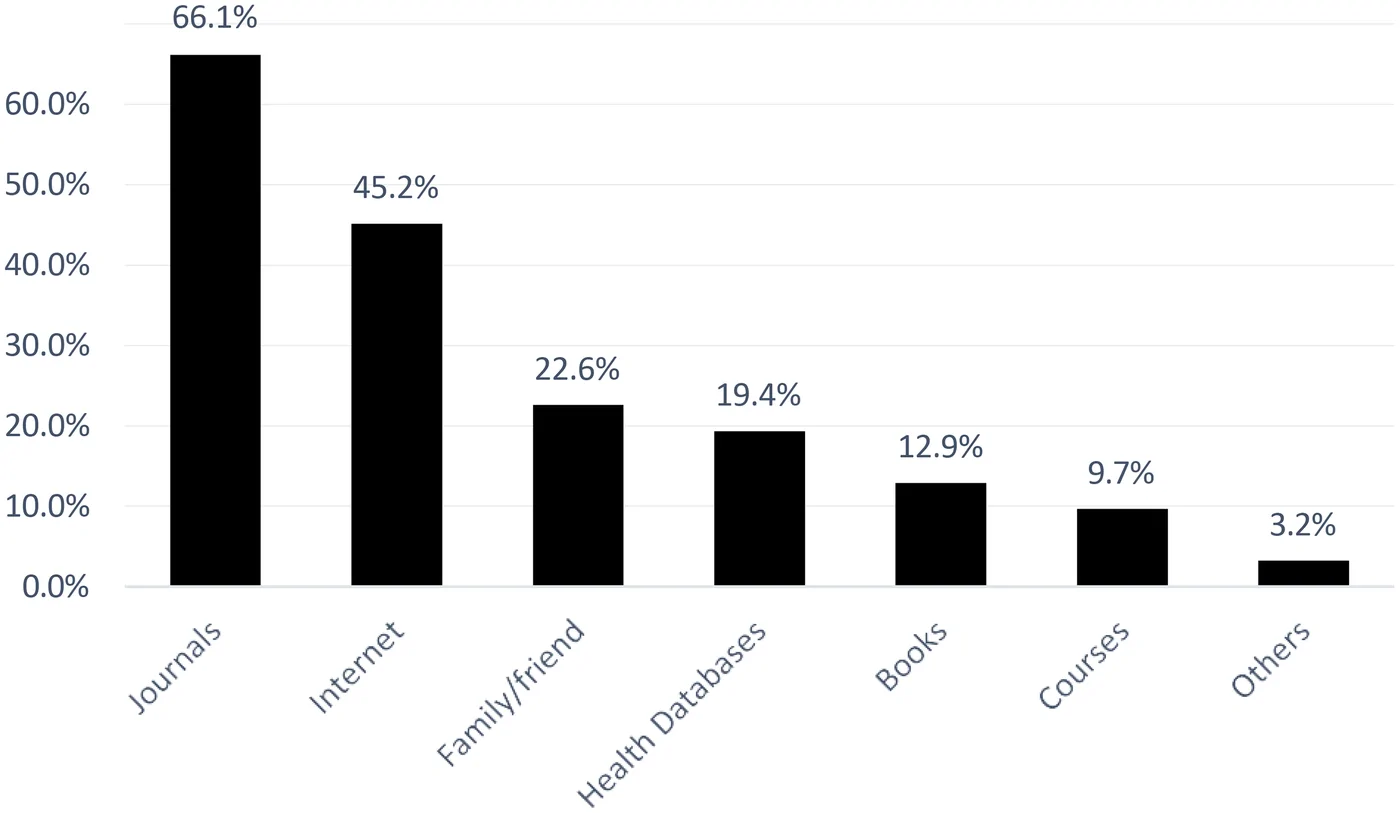
Reported sources of CAM knowledge among participating family physicians (*N* = 62). Participants could select more than one source; therefore, percentages do not sum to 100%. Bars are ordered from the most to the least frequently reported source.

Table 4 shows participating family physicians' generally positive attitudes toward CAM. Over half of participants (51.6%) viewed herbal products as a valid form of drug therapy and agreed that physicians with CAM knowledge benefit patients (64.5%). Strong support was reported for integrating evidence-based CAM into conventional care (79%), for stronger scientific evaluation of CAM therapies (72.6%), and for greater inclusion of CAM education in undergraduate medical curricula (82.2%). Despite this support, concerns about CAM posing a threat to public health remained (51.6%). However,CAM was perceived as popular in Saudi Arabia by 69.3% of participating physicians, while 62.9% supported its availability within the Ministry of Health budget.

**Table 4:** Attitudes of family physicians toward CAM (*N* = 62).

Attitude items	Strongly disagree		Disagree		Neutral		Agree		Strongly agree	
	*N*	%	*N*	%	*N*	%	*N*	%	*N*	%
The use of herbal products is a valid form of drug therapy that can be used in the treatment of a variety of diseases.	5	8.1%	6	9.7%	19	30.6%	17	27.4%	15	24.2%
Patients mostly benefit from physicians with some knowledge of complementary and alternative medicine.	3	4.8%	2	3.2%	17	27.4%	24	38.7%	16	25.8%
complementary and alternative medicine is a useful complement to pharmacological medicine.	4	6.5%	3	4.8%	16	25.8%	22	35.5%	17	27.4%
Complementary and alternative medicine can produce longer-lasting and more complete clinical results than conventional medicine.	5	8.1%	10	16.1%	21	33.9%	14	22.6%	12	19.4%
Complementary and alternative medicine should only be used as a last resort when conventional medicine has nothing to offer.	4	6.5%	14	22.6%	15	24.2%	14	22.6%	15	24.2%
Complementary and alternative medicine should only be used in minor ailments and not in the treatment of more serious illnesses.	2	3.2%	9	14.5%	10	16.1%	23	37.1%	18	29.0%
Incorporation of evidence-based complementary and alternative medicine therapies into conventional medicine practice would result in increased patient satisfaction.	2	3.2%	1	1.6%	10	16.1%	28	45.2%	21	33.9%
The results of complementary and alternative medicine are usually due to the placebo effect.	3	4.8%	9	14.5%	15	24.2%	20	32.3%	15	24.2%
CAM practices are not tested in a scientifically recognized manner (CAM therapies should be subject to more scientific testing before they can be accepted by conventional doctors)	2	3.2%	2	3.2%	13	21.0%	21	33.9%	24	38.7%
Complementary and alternative medicine should be made available within the current MOH budget.	2	3.2%	4	6.5%	17	27.4%	23	37.1%	16	25.8%
Doctors should receive more education about complementary and alternative medicine in undergraduate education.	1	1.6%	1	1.6%	9	14.5%	24	38.7%	27	43.5%
Complementary and alternative medicine is a threat to public health.	5	8.1%	12	19.4%	13	21.0%	18	29.0%	14	22.6%
Complementary and alternative medicine is popular in Saudi Arabia	1	1.6%	2	3.2%	16	25.8%	24	38.7%	19	30.6%

Together, beliefs and perceived effectiveness shaped attitudes, as shown in Table 5. More than half of participants (58.1%) reported the therapeutic effects of CAM, and most (75.8%) reported personal or professional experiences with CAM, with outcomes mainly described as beneficial (68.1%). Belief-related findings indicated that CAM practices were considered important (80.6%) and were associated with positive attitudes toward their use (74.2%). Over half (61.3%) believed CAM to be generally safe, with few side effects, although some participants remained uncertain or disagreed.

**Table 5:** Family physicians’ attitudes toward CAM are shaped by their beliefs and perceptions of its effectiveness. (*N* = 62).

Items	No	%
Have you seen the therapeutic effects of complementary and alternative medicine in your patients?		
Yes	36	58.1%
No	12	19.4%
Not sure	14	22.6%
Do you have any previous experience of complementary and alternative medicine?		
Yes	47	75.8%
No	15	24.2%
Not sure	0	0.0%
If the answer is yes, what were the outcomes?		
Beneficial	32	68.1%
Harmful	2	4.3%
Neither beneficial nor harmful	13	27.7%
Do you believe complementary and alternative medicine practices are important?		
Yes	50	80.6%
No	3	4.8%
Not sure	9	14.5%
Do you believe complementary and alternative medicine is safe and has few side effects?		
Yes	38	61.3%
No	10	16.1%
Not sure	14	22.6%
Do you have positive attitudes toward complementary and alternative medicine?		
Yes	46	74.2%
No	5	8.1%
Not sure	11	17.7%

Table 6 summarises clinical practice patterns related to CAM and patient care, showing that most participants suggested CAM therapies (79%) mainly to 0%–25% of patients per week (61.3%). The most commonly recommended modalities were meditation and relaxation techniques (64.5%), herbal and dietary supplements (59.7%), spiritual healing and prayers (58.1%), and cupping (53.2%), while less frequently reported modalities were recommended by fewer participants. Most respondents believed CAM to be suitable for both physical and psychological illnesses (74.2%), particularly hypertension, respiratory tract diseases, diabetes mellitus, and inflammatory conditions. Most participants reported discussing CAM with their patients (75.8%), while 80.6% addressed the potential harmful effects of specific CAM therapies, indicating frequent physician–patient communication regarding CAM use and its potential risks.

**Table 6:** Clinical practice patterns related to CAM among family physicians (*N* = 62).

Items		*N*	%
Do you suggest complementary and alternative medicine therapies to your patients?	Yes	49	79.0%
	No	13	21.0%
The percentage of patients you recommend complementary and alternative medicine per week	No	38	61.3%
	No	20	32.3%
	51–75%	4	6.5%
	Meditation/Hypnosis/Relaxation/Imagery	40	64.5%
	Herbal/Botanical/Dietary Supplements	37	59.7%
	Spiritual healing/prayers	36	58.1%
	Cupping	33	53.2%
	Massage	27	43.5%
	Yoga	13	21.0%
Complementary and alternative medicine products/therapies would you recommend	Reflexology	8	12.9%
	Homeopathy	7	11.3%
	Acupuncture	7	11.3%
	Naturopathy	7	11.3%
	Cauterization	7	11.3%
	Chiropractic	4	6.5%
	Aromatherapy	4	6.5%
	Others	5	8.0%
To which types of illness do you think complementary and alternative medicine is best suited?	Physical illness	6	9.7%
	Psychological illness	4	6.5%
	Both of them	46	74.2%
	Not sure	6	9.7%
	HTN	43	69.4%
	Respiratory tract-related diseases	39	62.9%
What are the common diseases patients prefer to use complementary and alternative medicine for?	DM	35	56.5%
	Inflammation	33	53.2%
	Wounds	32	51.6%
	Infection	31	50.0%
	Other	13	21.0%
Do you discuss complementary and alternative medicine with your patients?	Yes	47	75.8%
	No	15	24.2%
Do you discuss the possible harmful outcomes of specific complementary and alternative medicine treatments with them?	Yes	50	80.6%
	No	12	19.4%
	Never	3	4.8%
How often do your patients use complementary and alternative medicine?	rarely	11	17.7%
	Moderately	22	35.5%
	Frequently	26	41.9%
Do your patients use complementary and alternative medicine more than conventional medicine?	Yes	33	53.2%
	No	13	21.0%
	Not sure	16	25.8%

Figure 3 summarises family physicians' familiarity with a wide range of CAM practices. High levels of familiarity were observed with cultural and religious practices. Spiritual healing and prayer emerged as the most familiar modalities, with 69.4% of participants reporting awareness, followed by cupping (69.4%), massage therapy (67.7%), and herbal and dietary supplements (64.5%). By contrast, therapies that are less routinely incorporated into local clinical practice demonstrated lower levels of familiarity. A considerable proportion of participants reported unfamiliarity with acupuncture, chiropractic care, homeopathy, aromatherapy, and reflexology, with rates ranging from 43.5% to 54.8%.

**Figure 3:**
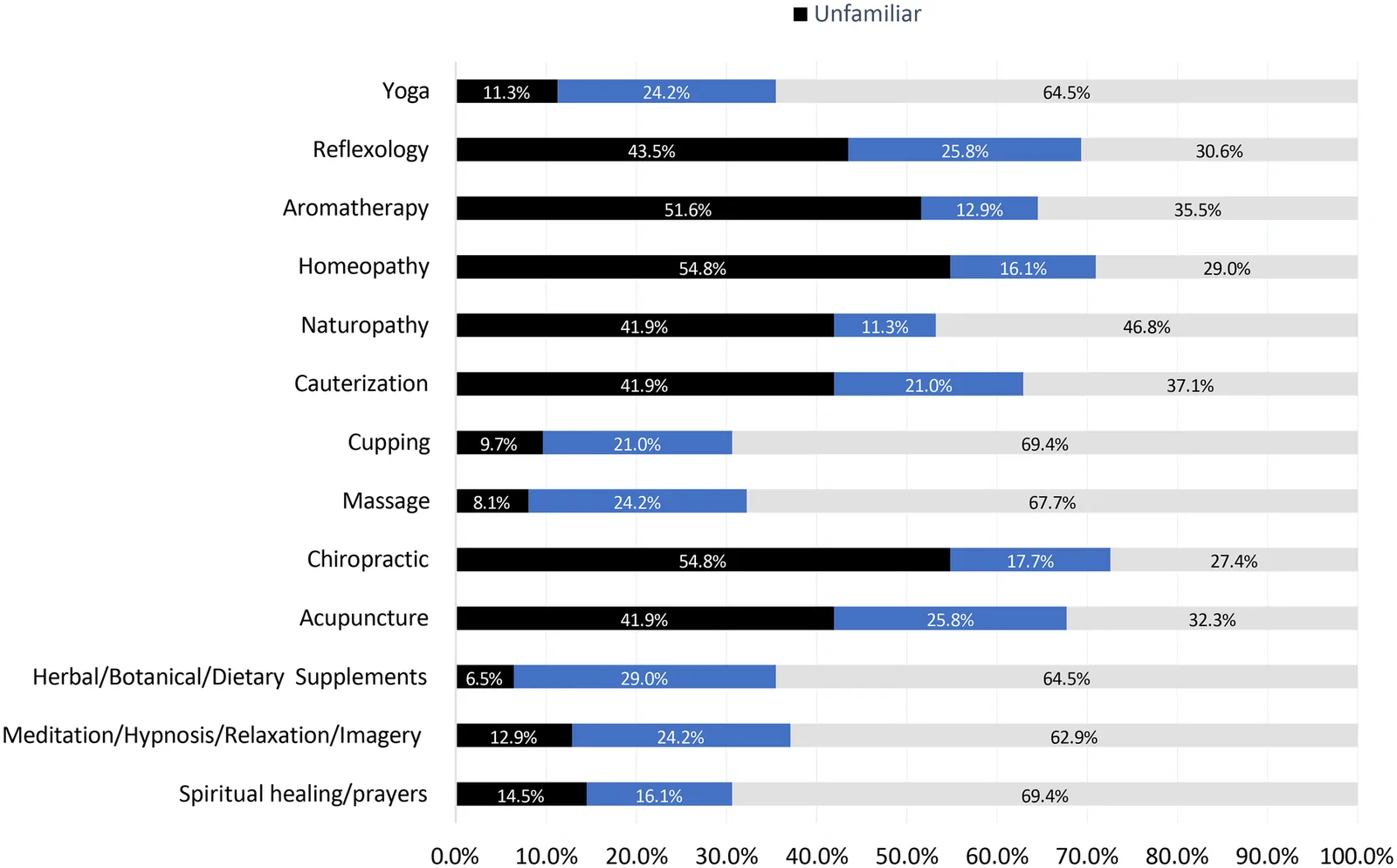
Familiarity with CAM modalities among participating family physicians (*N* = 62). Modalities are ordered from highest to lowest reported familiarity. Proportions are presented with Wilson 95% confidence intervals.

Table 7 examines factors associated with family physicians' overall knowledge of CAM. No statistically significant associations were observed between knowledge level and most sociodemographic or professional characteristics, including age group, gender, years of experience, professional classification, following CAM content on social media, or sources of CAM information (*p* > 0.05 for all). An association was observed between previous CAM training exposure and knowledge category. Participants who reported previous CAM training had a higher proportion of satisfactory knowledge than those without training exposure (50.0% vs. 28.6%; *p* = 0.049). Because the study was cross-sectional, this finding does not establish that training caused higher knowledge; physicians with greater prior interest in CAM may have been more likely to seek training, and residual confounding cannot be excluded.

**Table 7:** Factors associated with family physicians overall knowledge toward CAM.

Factors	Overall knowledge level				*p*-value
	Unsatisfactory		Satisfactory		
	No	%	No	%	
Age in years					
25–35	11	78.6%	3	21.4%	.451^
36–45	22	61.1%	14	38.9%	
46–55	7	58.3%	5	41.7%	
Gender					
Male	20	64.5%	11	35.5%	.999
Female	20	64.5%	11	35.5%	
Years of experiences					
< 5 years	11	73.3%	4	26.7%	.489
5–10 years	14	56.0%	11	44.0%	
> 10 years	15	68.2%	7	31.8%	
Professional classification					
GP	6	85.7%	1	14.3%	
Resident	5	62.5%	3	37.5%	.654^
Specialist	4	66.7%	2	33.3%	
Consultant	25	61.0%	16	39.0%	
CAM training exposure					
Yes	10	50.0%	10	50.0%	.049*
No	30	71.4%	12	28.6%	
CAM social media engagement					
Yes	19	57.6%	14	42.4%	.223
No	21	72.4%	8	27.6%	
Source of CAM information					
Internet	18	64.3%	10	35.7%	
Journals	25	61.0%	16	39.0%	
Health Databases	8	66.7%	4	33.3%	.914^
Family/friend	10	71.4%	4	28.6%	
Courses	4	66.7%	2	33.3%	
Books	6	75.0%	2	25.0%	
Others	2	100.0%	0	0.0%	

P: Pearson ×2 test ^: Exact Probability test *P < 0.05 (significant).

Table 8 examines the relationship between overall knowledge level of CAM and clinical practice patterns. Physicians who suggested CAM therapies were more likely to have satisfactory knowledge, although this association was not statistically significant (*p* = 0.293). Knowledge category was associated with the reported proportion of patients for whom CAM was recommended per week (*p* = 0.012). Participants classified as having satisfactory knowledge more frequently reported recommending CAM to larger proportions of their patients. Knowledge category was also associated with the type of illness considered suitable for CAM (*p* = 0.049). These findings represent exploratory cross-sectional associations and should not be interpreted as evidence that greater knowledge directly caused changes in recommendation behaviour. Other practices, including discussing CAM use and addressing potential harmful outcomes, were more common among those with satisfactory knowledge, though not statistically significant.

**Table 8:** Association between overall knowledge level and clinical practice patterns related to CAM among family physicians.

	Overall knowledge level				*p*-value
	Unsatisfactory		Satisfactory		
	No	%	No	%	
Do you suggest complementary and alternative medicine therapies to your patients?					.293
Yes	30	75.0%	19	86.4%	
No	10	25.0%	3	13.6%	
The percentage of patients you recommend complementary and alternative medicine per week					
0–25%	28	70.0%	10	45.5%	
26–50%	12	30.0%	8	36.4%	012*
51–75%	0	0.0%	4	18.2%	
To which types of illness do you think complementary and alternative medicine is best suited?					
Physical illness	1	2.5%	5	22.7%	.049*
Psychological illness	3	7.5%	1	4.5%	
Both of them	31	77.5%	15	68.2%	
None of them	5	12.5%	1	4.5%	
Do you discuss complementary and alternative medicine with your patients?					
Yes	28	70.0%	19	86.4%	.150
No	12	30.0%	3	13.6%	
Do you discuss the possible harmful outcomes of specific complementary and alternative medicine treatments with them?					.129
Yes	30	75.0%	20	90.9%	
No	10	25.0%	2	9.1%	

P: Exact Probability test; *P < 0.05.

## Discussion

This exploratory study describes family physicians' knowledge, attitudes, counselling confidence, and self-reported CAM-related practices in Saudi Arabia. The findings were broadly consistent with previous Saudi, Gulf, and international studies reporting generally positive attitudes toward evidence-based CAM integration, variable knowledge, limited formal training, and greater familiarity with culturally prevalent therapies. Accordingly, the contribution of this study is primarily contextual and incremental rather than methodological or theoretical. Its specific contribution lies in providing recent regional data from family physicians and in describing formal training exposure, counselling confidence concerning commonly used herbs, and recommendation patterns across individual CAM modalities.

### Knowledge of CAM among family physicians and comparison with previous literature

The results showed that most family physicians had an average level of overall CAM knowledge, with more than a third demonstrating satisfactory knowledge and only a small proportion having low knowledge levels. Although general knowledge of CAM concepts and modalities was high, self-assessed knowledge varied considerably, reflecting a widely reported global trend in which healthcare professionals are familiar with CAM in principle but lack a comprehensive understanding of specific therapies and their clinical applications. Similar findings have been documented among physicians and medical students in Singapore, the United Kingdom and North America, where knowledge of CAM is high but formal competence remains inconsistent (27–29).

Previous formal CAM training was associated with higher observed knowledge levels in this exploratory sample. However, this relationship should not be interpreted causally because the cross-sectional design cannot establish whether training preceded higher knowledge. It is also possible that physicians with greater pre-existing interest, familiarity, or confidence in CAM were more likely to seek formal training. Larger longitudinal or interventional studies are needed to determine whether structured CAM education produces measurable improvements in objective knowledge and clinical decision-making (22–24, 27, 28). In contrast, demographic and professional variables, such as age, gender, years of experience, professional classification, and engagement with CAM-related social media content, were not significantly associated with knowledge. This observed association is broadly consistent with previous studies reporting greater CAM knowledge among healthcare professionals with educational exposure; however, the present study cannot determine whether education itself accounted for the difference (30). The absence of an association between social media use and knowledge further raises concerns about the reliability of informal information sources for developing clinical confidence (31).

Participants demonstrated the greatest knowledge and counselling confidence for culturally embedded and commonly used herbs, including ginger, garlic, cinnamon, black seed, and turmeric. This pattern may reflect cultural exposure, patient demand, or familiarity. Conversely, herbs such as echinacea, ginseng, and ashwagandha despite their increasing global popularity were associated with lower counselling confidence, suggesting limited exposure within local medical education and clinical practice. This selective familiarity is consistent with findings from Middle Eastern and Asian contexts, where physicians tend to recommend therapies that are culturally normative and religiously accepted (25, 32). While such familiarity may facilitate patient–physician communication, it also highlights concerns about uneven evidence appraisal and emphasises the need for standardised, evidence-based CAM education (25).

Comparisons with international studies require cautious interpretation because differences in healthcare systems, educational frameworks, professional regulation, cultural context, access to integrative services, and study methodology can all influence reported knowledge and attitudes regarding CAM (6, 31). Observed variations may result from differences in questionnaires, scoring systems, participant characteristics, and sampling strategies, rather than reflecting genuine disparities in physician competence (7).

Support for evidence-based CAM integration, alongside concerns about potential public health risks, is not inherently contradictory (32). Physicians may differentiate between regulated, evidence-informed CAM and practices that are unregulated or insufficiently evaluated (28). Similarly, endorsement of CAM education may indicate a desire to enhance physicians’ capacity to critically appraise evidence, counsel patients, and manage potential interactions, rather than representing unconditional support for all CAM therapies (29). The underlying reasons for these perspectives were not investigated in this study and therefore remain speculative.

The findings indicate possible gaps between attitudes and practices, as well as between knowledge and practices. While physicians generally expressed support for evidence-based CAM integration, relatively few reported recommending CAM or having received formal training (19). This discrepancy may be attributed to barriers such as limited confidence, uncertainty regarding the evidence, and a lack of clinical guidance (8). Given that the cross-sectional design does not establish causality, longitudinal and qualitative studies are required to elucidate these relationships and the factors influencing CAM-related practices (32).

### Family physicians' attitudes toward CAM

In this study, family physicians demonstrated predominantly positive attitudes toward CAM, particularly when it was perceived as complementary rather than an alternative to conventional medical practice. There was strong endorsement of integrating evidence-based CAM therapies into routine clinical care, alongside support for enhanced scientific evaluation of CAM and for including CAM education in undergraduate medical curricula. However, a substantial proportion of participants viewed CAM as a potential public health concern, suggesting a measured critical perspective rather than uncritical acceptance. This cautious optimism is consistent with findings from studies conducted in Europe and North America, where healthcare professionals generally support CAM integration but stress the importance of regulation, robust scientific evidence, and patient safety (33–35).

Participants largely agreed that physicians with CAM knowledge are better equipped to benefit patients, and many emphasised the value of integrating CAM within conventional practice frameworks. These attitudes appear to be shaped by perceived therapeutic benefits, cultural acceptability, and clinical experience, as reported in previous research (35). Notably, participants expressed reservations about CAM's superiority over conventional medicine and its use in the management of serious illnesses, reflecting an evidence-based scepticism observed in other studies. Furthermore, the widespread recognition of the need for formal CAM education at the undergraduate level aligns with global recommendations to incorporate CAM competencies into medical education curricula (36).

### Practice patterns and patient engagement

Most participants reported actively recommending CAM therapies, although typically to a limited proportion of their patients. Mind–body interventions, herbal and dietary supplements, spiritual healing, and cupping were the most frequently recommended modalities possibly reflecting cultural familiarity and perceived acceptability. In contrast, less culturally integrated or less familiar practices, such as acupuncture, chiropractic therapy, homoeopathy, and aromatherapy, were recommended less often.

Encouragingly, most physicians reported discussing CAM use with patients and addressing potential adverse effects, indicating responsible clinical engagement. This is particularly important in Saudi Arabia, where CAM use is widespread and frequently occurs alongside conventional treatment. The high frequency of patient CAM use reported by physicians further underscores the need for active physician involvement to prevent harmful interactions and support informed decision-making (15, 32).

As for the knowledge–practice relationship, although simply recommending CAM was not significantly associated with knowledge level, the proportion of patients receiving CAM recommendations and the types of conditions for which CAM was considered appropriate were significantly associated with higher knowledge. Physicians with satisfactory knowledge were more likely to recommend CAM to a greater proportion of patients and to recognise its applicability to physical illnesses. The observed association between knowledge and self-reported CAM recommendation patterns should be interpreted cautiously, as this study did not assess the appropriateness, safety, evidence base, or clinical outcomes of individual recommendations. Consequently, it cannot determine whether greater knowledge leads to more appropriate CAM integration (28). Furthermore, evidence-based CAM integration should not be equated with unconditional acceptance. Safe integration requires critical appraisal of the evidence, appropriate clinical assessment, documentation, safety monitoring, and ongoing evaluation to minimise potential risks, including delayed conventional treatment, adverse effects, and herb–drug interactions (23, 27).

### Clinical, public health, and research implications

The findings establish priorities for future research rather than providing justification for immediate policy or practice changes. Subsequent studies should investigate physicians' access to evidence-based CAM resources, documentation and medication reconciliation practices, as well as training in evidence appraisal, herb–drug interactions, contraindications, patient counselling, and referral. Potential educational strategies comprise competency-based undergraduate and continuing professional development programs, supported by evidence-based clinical resources and standardized documentation. However, the feasibility, effectiveness, safety, and cost-effectiveness of these strategies should be rigorously evaluated prior to implementation. It is important to note that this study did not assess the appropriateness, safety, or clinical outcomes of physicians' CAM recommendations. Consequently, the findings should not be interpreted as evidence that CAM training enhances patient outcomes or that the reported recommendations were evidence-based. While familiarity with culturally accepted CAM practices may facilitate patient communication, it does not equate to evidence-based knowledge or clinical competence.

Medical education should emphasize the development of competencies in critically appraising CAM evidence, identifying herb–drug and supplement–drug interactions, recognizing adverse effects and contraindications, conducting medication reconciliation, understanding regulatory frameworks, and communicating effectively with patients regarding CAM use. These competencies may be integrated through case-based learning, instruction in clinical pharmacology and medication safety, simulated counseling scenarios, and supervised clinical discussions with patients who use CAM in conjunction with conventional therapies. Educational approaches should prioritize evidence-informed decision-making rather than unconditional acceptance or rejection of CAM.

Future research should employ larger, multicentre studies with representative samples and validated, objective measures of CAM knowledge and practice. Prospective, interventional, and qualitative studies are necessary to assess the impact of CAM education, identify barriers to evidence-based integration, and elucidate the gaps between knowledge and practice as well as attitude and practice. Patient-centred research should further investigate the effects of physician knowledge on CAM disclosure, shared decision-making, safety, clinical outcomes, and the systematic evaluation of both culturally familiar and less familiar CAM therapies.

### Strengths and limitations

This study provides preliminary evidence regarding family physicians' knowledge, attitudes, counselling confidence, and self-reported practices related to complementary and alternative medicine (CAM) in Saudi Arabia. Several limitations warrant consideration. The cross-sectional design precludes causal inference, and the small, non-probability sample limits statistical power and generalisability. Potential selection and non-response biases may have influenced participation, while reliance on self-reported measures introduces susceptibility to recall and social desirability biases. Furthermore, interviewer administration, limited psychometric validation of the questionnaire, and the operationalisation of knowledge classifications may have contributed to measurement error.

The exploratory analyses involved multiple comparisons without multivariable adjustment due to the limited sample size, increasing the risk of false-positive findings and residual confounding. The study did not evaluate the appropriateness, safety, evidence base, or clinical outcomes of the reported CAM recommendations. Consequently, these results should be interpreted as preliminary descriptions of participating physicians' reported knowledge, attitudes, counselling confidence, and practices, rather than as evidence supporting effective or appropriate CAM integration. Larger, nationally representative, longitudinal studies employing validated instruments and objective measures are required to substantiate these findings.

The cultural and religious context of Saudi Arabia exerts a significant influence on CAM practices, especially herbal remedies, cupping, and spiritual healing. In this exploratory study, physicians' increased familiarity with these practices likely results from cultural exposure and patient interactions rather than from evidence-based clinical competence. Although cultural familiarity can enhance communication and promote disclosure of CAM use, it may also foster unsubstantiated assumptions regarding safety or efficacy. Consequently, culturally sensitive CAM counselling should acknowledge patients' beliefs while upholding evidence-based practice and prioritizing patient safety, including careful assessment of adverse effects, contraindications, and herb–drug interactions.

## Conclusion

This exploratory cross-sectional study identified that family physicians possessed moderate knowledge of CAM, demonstrated generally positive attitudes toward evidence-based CAM, and tended to recommend therapies that were culturally familiar. Given the limited regional sample and the study's design, these findings require cautious interpretation. Further multicentre research employing validated objective measures is necessary to confirm these results and to evaluate their implications for clinical practice and medical education.

## Data Availability

The original contributions presented in the study are included in the article/Supplementary Material, further inquiries can be directed to the corresponding author/s.
