# Supplementary material for: Family physicians’ knowledge, attitudes, and practices regarding complementary and alternative medicine in Saudi Arabia: an exploratory cross-sectional study
Source: Front Health Serv. 2026 Oct 7;6:1837145. doi: 10.3389/frhs.2026.1837145 (PMC13683619; doi:10.3389/frhs.2026.1837145)
Supplement: Supplementary file 1 [file Table1.docx]

**Supplementary Material S1**

**Study Questionnaire**

# **Section A: Sociodemographic and Professional Characteristics**

**A1.** Age group

☐ 25–35 years	☐ 36–45 years	☐ 46–55 years

**A2.** Gender

☐ Male	☐ Female

**A3.** Years of professional experience

☐ < 5 years	☐ 5–10 years	☐ > 10 years

**A4.** Professional classification

☐ General practitioner	☐ Resident	☐ Specialist	☐ Consultant

**A5.** Have you attended any formal CAM training (e.g., lectures, workshops, or certification programs)?

☐ Yes	☐ No

**A6.** Do you follow CAM-related content on social media?

☐ Yes	☐ No

# **Section B: Knowledge of Complementary and Alternative Medicine (CAM)**

**B1.** Do you know what the abbreviation CAM stands for?

☐ Yes	☐ No

**B2.** Have you heard of complementary and alternative medicine before?

☐ Yes	☐ No

**B3.** How would you rate your own knowledge of CAM therapies?

☐ None	☐ Limited	☐ Moderate	☐ Excellent

**B4.** Do you think CAM should be an integral part of conventional medicine?

☐ Yes	☐ No	☐ Not sure

**B5.** In your opinion, what is the preferred role of CAM in relation to conventional medicine?

☐ A complement to conventional medicine	☐ An integral part of conventional medicine	☐ An alternative to conventional medicine	☐ Not sure

**B6.** Are you aware of clear national guidelines regulating CAM practice in Saudi Arabia?

☐ Yes	☐ No	☐ Not sure

**B7. For each of the following commonly used herbs, please indicate your level of knowledge and your confidence in counselling patients about its medicinal use, benefits, and risks:**

| **Herb** | **Not familiar; not confident counselling patients** | **Familiar; not confident counselling patients** | **Familiar; confident counselling patients** |
| --- | --- | --- | --- |
| Ginger | ☐ | ☐ | ☐ |
| Garlic | ☐ | ☐ | ☐ |
| Cinnamon | ☐ | ☐ | ☐ |
| Fenugreek | ☐ | ☐ | ☐ |
| Coriander | ☐ | ☐ | ☐ |
| Ginseng | ☐ | ☐ | ☐ |
| Echinacea | ☐ | ☐ | ☐ |
| Ashwagandha | ☐ | ☐ | ☐ |
| Black seed (Nigella sativa) | ☐ | ☐ | ☐ |
| Turmeric | ☐ | ☐ | ☐ |
| Roselle | ☐ | ☐ | ☐ |

# **Section C: Attitudes toward CAM**

| **No.** | **Statement** | **Strongly disagree** | **Disagree** | **Neutral** | **Agree** | **Strongly agree** |
| --- | --- | --- | --- | --- | --- | --- |
| 1 | The use of herbal products is a valid form of drug therapy that can be used in the treatment of a variety of diseases. | ☐ | ☐ | ☐ | ☐ | ☐ |
| 2 | Patients mostly benefit from physicians with some knowledge of complementary and alternative medicine. | ☐ | ☐ | ☐ | ☐ | ☐ |
| 3 | Complementary and alternative medicine is a useful complement to pharmacological medicine. | ☐ | ☐ | ☐ | ☐ | ☐ |
| 4 | Complementary and alternative medicine can produce longer-lasting and more complete clinical results than conventional medicine. | ☐ | ☐ | ☐ | ☐ | ☐ |
| 5 | Complementary and alternative medicine should only be used as a last resort when conventional medicine has nothing to offer. | ☐ | ☐ | ☐ | ☐ | ☐ |
| 6 | Complementary and alternative medicine should only be used in minor ailments and not in the treatment of more serious illnesses. | ☐ | ☐ | ☐ | ☐ | ☐ |
| 7 | Incorporation of evidence-based complementary and alternative medicine therapies into conventional medicine practice would result in increased patient satisfaction. | ☐ | ☐ | ☐ | ☐ | ☐ |
| 8 | The results of complementary and alternative medicine are usually due to the placebo effect. | ☐ | ☐ | ☐ | ☐ | ☐ |
| 9 | CAM practices are not tested in a scientifically recognised manner (CAM therapies should be subject to more scientific testing before they can be accepted by conventional doctors). | ☐ | ☐ | ☐ | ☐ | ☐ |
| 10 | Complementary and alternative medicine should be made available within the current Ministry of Health (MOH) budget. | ☐ | ☐ | ☐ | ☐ | ☐ |
| 11 | Doctors should receive more education about complementary and alternative medicine in undergraduate education. | ☐ | ☐ | ☐ | ☐ | ☐ |
| 12 | Complementary and alternative medicine is a threat to public health. | ☐ | ☐ | ☐ | ☐ | ☐ |
| 13 | Complementary and alternative medicine is popular in Saudi Arabia. | ☐ | ☐ | ☐ | ☐ | ☐ |

**Beliefs and perceived effectiveness**

**C1.** Have you seen the therapeutic effects of complementary and alternative medicine in your patients?

☐ Yes	☐ No	☐ Not sure

**C2.** Do you have any previous personal or professional experience of complementary and alternative medicine?

☐ Yes	☐ No	☐ Not sure

*If yes, what were the outcomes? ☐ Beneficial	☐ Harmful	☐ Neither beneficial nor harmful*

**C3.** Do you believe complementary and alternative medicine practices are important?

☐ Yes	☐ No	☐ Not sure

**C4.** Do you believe complementary and alternative medicine is safe and has few side effects?

☐ Yes	☐ No	☐ Not sure

**C5.** Overall, do you have positive attitudes toward complementary and alternative medicine?

☐ Yes	☐ No	☐ Not sure

# **Section D: Clinical Practices Related to CAM**

**D1.** Do you discuss or recommend CAM therapies to your patients?

☐ Yes	☐ No

**D2.** If yes, to approximately what proportion of your patients per week do you recommend CAM?

☐ 0–25%	☐ 26–50%	☐ 51–75%	☐ 76–100%

**D3.** Which of the following CAM modalities do you recommend to your patients? (Select all that apply)

- ☐ Meditation / hypnosis / relaxation / imagery
- ☐ Herbal and dietary supplements
- ☐ Spiritual healing / prayers
- ☐ Cupping (Hijama)
- ☐ Massage therapy
- ☐ Acupuncture
- ☐ Chiropractic therapy
- ☐ Naturopathy
- ☐ Cauterization
- ☐ Homeopathy
- ☐ Aromatherapy
- ☐ Reflexology
- ☐ Yoga
- ☐ Other (please specify): ____________

**D4.** For which of the following clinical conditions do you consider CAM appropriate? (Select all that apply)

- ☐ Hypertension
- ☐ Respiratory tract diseases
- ☐ Diabetes mellitus
- ☐ Inflammatory conditions
- ☐ Psychological / emotional conditions
- ☐ Other (please specify): ____________
- ☐ None

**D5.** In general, do you consider CAM to be suitable for physical illnesses, psychological illnesses, or both?

☐ Physical illnesses only	☐ Psychological illnesses only	☐ Both	☐ Neither

**D6.** Do you discuss the potential harms or risks of CAM with your patients?

☐ Yes, always	☐ Sometimes	☐ No

**D7.** Do you ask your patients whether they are using CAM alongside their conventional medicine treatments?

☐ Yes, always	☐ Sometimes	☐ No

**D8.** In your experience, how frequently do your patients use CAM relative to conventional medicine?

☐ Never	☐ Rarely	☐ Sometimes	☐ Often	☐ More than conventional medicine

**D9. For each of the following CAM modalities, please indicate whether you are familiar or unfamiliar with it:**

| **CAM modality** | **Familiar** | **Unfamiliar** |
| --- | --- | --- |
| Spiritual healing / prayers | ☐ | ☐ |
| Meditation / hypnosis / relaxation / imagery | ☐ | ☐ |
| Herbal / botanical / dietary supplements | ☐ | ☐ |
| Acupuncture | ☐ | ☐ |
| Chiropractic therapy | ☐ | ☐ |
| Massage therapy | ☐ | ☐ |
| Cupping (Hijama) | ☐ | ☐ |
| Cauterization | ☐ | ☐ |
| Naturopathy | ☐ | ☐ |
| Homeopathy | ☐ | ☐ |
| Aromatherapy | ☐ | ☐ |
| Reflexology | ☐ | ☐ |
| Yoga | ☐ | ☐ |
