# Supplementary material for: Family physicians’ knowledge, attitudes, and practices regarding complementary and alternative medicine in Saudi Arabia: an exploratory cross-sectional study
Source: Front Health Serv. 2026 Oct 7;6:1837145. doi: 10.3389/frhs.2026.1837145 (PMC13683619; doi:10.3389/frhs.2026.1837145)
Supplement: Supplementary file 2 [file Table2.docx]

**Supplementary Material S2**

**STROBE Statement — Checklist of Items for Cross-Sectional Studies**

| **Item No.** | **Recommendation** | **Reported** | **Location in manuscript** |
| --- | --- | --- | --- |
| **Title and abstract** | | | |
| 1a | Indicate the study's design with a commonly used term in the title or the abstract. | Yes | Page 1 |
| 1b | Provide in the abstract an informative and balanced summary of what was done and what was found. | Yes | Page 4 |
| **Introduction** | | | |
| 2 | Background/rationale: Explain the scientific background and rationale for the investigation being reported. | Yes | Page 4,5 |
| 3 | Objectives: State specific objectives, including any prespecified hypotheses. | Yes | Page 5 |
| **Methods** | | | |
| 4 | Study design: Present key elements of study design early in the paper. | Yes | Page 6 |
| 5 | Setting: Describe the setting, locations, and relevant dates, including periods of recruitment, exposure, follow-up, and data collection. | Yes | Page 6 |
| 6 | Participants: Give the eligibility criteria, and the sources and methods of selection of participants. | Yes | Page 6 |
| 7 | Variables: Clearly define all outcomes, exposures, predictors, potential confounders, and effect modifiers. Give diagnostic criteria, if applicable. | Yes | Page 6,7, Supplementary Material 1 |
| 8 | Data sources/measurement: For each variable of interest, give sources of data and details of methods of assessment (measurement). Describe comparability of assessment methods if there is more than one group. | Yes | Page 7, Supplementary Material 1 |
| 9 | Bias: Describe any efforts to address potential sources of bias. | Yes | Page 7 |
| 10 | Study size: Explain how the study size was arrived at. | Yes | Page 6,7,24 |
| 11 | Quantitative variables: Explain how quantitative variables were handled in the analyses. If applicable, describe which groupings were chosen and why. | Yes | Page 7,8 |
| 12a–e | Statistical methods: (a) methods used to control for confounding; (b) subgroup/interaction analyses; (c) handling of missing data; (d) sampling strategy, if applicable; (e) sensitivity analyses. | Yes | Page 7,8,24 |
| **Results** | | | |
| 13a–c | Participants: (a) Numbers of individuals at each stage of the study; (b) reasons for non-participation; (c) consider a flow diagram. | Yes | Page 8 |
| 14a–c | Descriptive data: (a) Characteristics of participants and information on exposures/potential confounders; (b) number of participants with missing data; (c) follow-up time (if applicable). | Yes | Page 8,9 |
| 15 | Outcome data: Report numbers of outcome events or summary measures. | Yes | Page 9–18 |
| 16a–c | Main results: (a) Unadjusted and, if applicable, adjusted estimates and precision (e.g., 95% CI); (b) category boundaries for categorised continuous variables; (c) translation of relative risk to absolute risk where relevant. | Yes | Tables 7–8, Page 7,8, Page 18-20 |
| 17 | Other analyses: Report other analyses done—e.g., subgroup and interaction analyses, and sensitivity analyses. | Yes | Tables 7–8, Page 18-20 |
| **Discussion** | | | |
| 18 | Key results: Summarise key results with reference to study objectives. | Yes | Page 21 |
| 19 | Limitations: Discuss limitations of the study, taking into account sources of potential bias or imprecision. Discuss both direction and magnitude of any potential bias. | Yes | Page 24 |
| 20 | Interpretation: Give a cautious overall interpretation considering objectives, limitations, multiplicity of analyses, results from similar studies, and other relevant evidence. | Yes | Page 21-24 |
| 21 | Generalisability: Discuss the generalisability (external validity) of the study results. | Yes | Page 1, 24 |
| **Other information** | | | |
| 22 | Funding: Give the source of funding and the role of the funders for the present study and, if applicable, for the original study on which the present article is based. | Yes | Page 3 |
